# Twenty-Years of Experience in Childhood Glaucoma Surgery

**DOI:** 10.3390/jcm10245720

**Published:** 2021-12-08

**Authors:** Felix Mathias Wagner, Alexander Karl-Georg Schuster, Franz Grehn, Lukas Urbanek, Norbert Pfeiffer, Julia Verena Stingl, Esther Maria Hoffmann

**Affiliations:** Department of Ophthalmology, University Medical Center Mainz, Langenbeckstr. 1, 55131 Mainz, Germany; felix.wagner@unimedizin-mainz.de (F.M.W.); alexander.schuster@uni-mainz.de (A.K.-G.S.); Grehn_F@ukw.de (F.G.); l.urbanek@hotmail.de (L.U.); Norbert.Pfeiffer@unimedizin-mainz.de (N.P.); julia.stingl@unimedizin-mainz.de (J.V.S.)

**Keywords:** glaucoma, childhood glaucoma, glaucoma surgery

## Abstract

To quantify the results of childhood glaucoma treatment over time in a cohort of children with different types of childhood glaucoma. A retrospective cohort study of consecutive cases involving children with primary congenital glaucoma, primary juvenile, and secondary juvenile glaucoma at the Childhood Glaucoma Center, University Medical Center Mainz, Germany from 1995 to 2015 was conducted. The main outcome measure was the long-term development of intraocular pressure. Further parameters such as surgical success, refraction, corneal diameter, axial length, and surgical procedure in children with different types of childhood glaucoma were evaluated. Surgical success was defined as IOP < 21 mmHg in eyes without a need for further intervention for pressure reduction. A total of 93 glaucomatous eyes of 61 childhood glaucoma patients with a mean age of 3.7 ± 5.1 years were included. The overall mean intraocular pressure at first visit was 32.8 ± 10.2 mmHg and decreased to 15.5 ± 7.3 mmHg at the last visit. In the median follow-up time of 78.2 months, 271 surgical interventions were performed (130 of these were cyclophotocoagulations). Many (61.9%) of the eyes that underwent surgery achieved complete surgical success without additional medication. Qualified surgical success (with or without additional medication) was reached by 84.5% of the eyes.

## 1. Introduction

Childhood glaucoma is a heterogeneous group of disorders that each require careful attention and understanding to prevent a lifetime of vision loss. It is characterized by intraocular pressure (IOP)-related damage to the eye and is caused by a diverse group of conditions [1,2]. The Childhood Glaucoma Research Network (CGRN) defined the following classification system for childhood glaucoma: primary glaucoma including juvenile open-angle glaucoma and primary congenital glaucoma and secondary childhood glaucoma including glaucoma following cataract surgery, glaucoma associated with nonacquired systemic disease or syndrome, glaucoma associated with nonacquired ocular anomalies, and glaucoma associated with acquired conditions [1].

Primary childhood glaucoma and secondary childhood glaucoma due to other ocular anomalies can lead to severe visual impairment and even blindness if not treated. While each of these disorders is very rare, primary congenital glaucoma (PCG) is among the most frequent [3,4]. The incidence of PCG differs between regions. In the Western world, the incidences range between 1:10,000 and 1:30,000 [3,4]. However, the incidences increase in populations with higher proportion of consanguinity [5].

Notably, the elevated IOP common to all forms of childhood glaucoma can lead to stretching of the globe’s outer coats, producing corneal enlargement, scleral thinning, and axial length growth, thus leading to myopic shifts and possible anisometropia. Myopia is highly associated with childhood glaucoma and should therefore be taken into account, together with the axial length, in follow-up examinations of patients with childhood glaucoma [6]. If not treated early on, anisometropia due to a refractive change in glaucomatous eyes may lead to stimulus deprivation amblyopia, which has been reported to be one of the major causes of vision loss in these children, up to almost 50% [7].

Successful control of IOP and axial length growth is therefore crucial in the management of these conditions, along with ametropia correction and amblyopia treatment, to optimize long-term visual outcomes. Medical therapy plays a minor role in controlling IOP, except in secondary glaucoma. Surgery is the first-line treatment, especially in PCG, where angle surgery is accepted as a state-of-the-art treatment [3,8,9].

Due to its rare occurrence, there are only a few small studies and case series on the surgical treatment of childhood glaucoma. Opening of the trabecular meshwork and the Schlemm’s canal is the preferred surgical approach, such as probe trabeculotomy, 360°-trabeculotomy, goniotomy, and trabeculectomy [10]. 

Trabeculotomy has the advantage of feasibility even with a cloudy cornea, and a 360° surgery of the angle is possible in a single procedure [11,12,13,14,15]. The success rates lie between 60 and 87% for the classic trabeculotomy in a follow-up period of 1–3 years [13]. The success rates of 360° trabeculotomy are between 72 and 92% in a follow-up period of 1–4 years [16]. They are particularly high if the children are operated on within the first year of life [13]. Possible complications are the presence of hyphema (although an expected result of surgery), choroidal detachment, descemetolysis, and damage of the iris or of the lens [17].

The aim of this work was to quantify the results of childhood glaucoma treatment over time, including all its different forms due to their rare occurrence. Parameters such as refraction, corneal diameter, axial length, intraocular pressure, and surgical procedure in children with different types of childhood glaucoma were evaluated at the Childhood Glaucoma Center of the University Medical Center of Mainz between the years 1995 and 2015.

## 2. Materials and Methods

**Figure 1:**
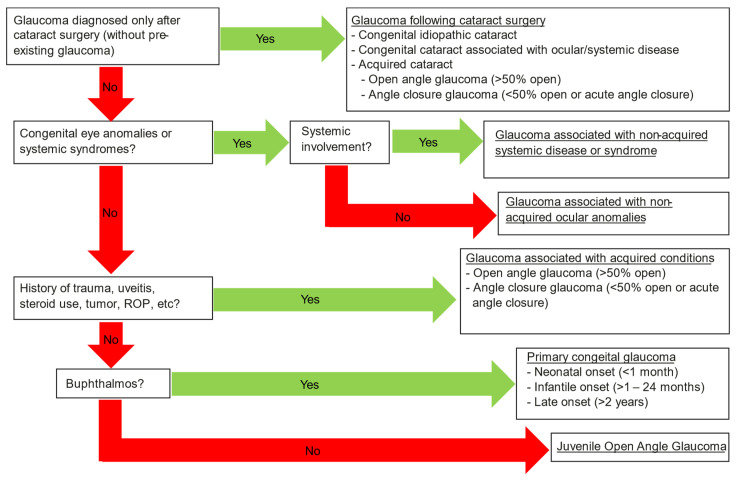
Childhood glaucoma categorization according to the Childhood Glaucoma Research Network classification [1].

This is a single-institution retrospective cohort study involving children with childhood glaucoma (primary congenital glaucoma (PCG), primary juvenile, and secondary juvenile glaucoma) at the Department of Ophthalmology of the University Medical Center of Mainz, from 1995 to 2015. Patients diagnosed with childhood glaucoma were included if they had glaucoma surgery between 1995 and 2015 and did not exceed the age of 18 years. Childhood glaucoma was defined according to the 9th Consensus Report of the World Glaucoma Association and further by the Childhood Glaucoma Research Network (CGRN) classification system for childhood glaucoma (Figure 1) [1,2]. Patients younger than 18 years of age, IOP > 21 mmHg, and glaucomatous optic nerve head changes without any history or signs of ocular defects were considered primary childhood glaucoma (according to the above-mentioned criteria) [1]. If ocular or systematic anomalies were responsible for elevated IOP, patients were considered to have secondary childhood glaucoma. The group with secondary childhood glaucoma was divided into glaucoma following cataract surgery (aphakic glaucoma); glaucoma associated with nonacquired systemic disease or syndrome (e.g., Sturge-Weber syndrome); glaucoma associated with nonacquired ocular anomalies (e.g., Peter’s anomaly Axenfeld-Rieger syndrome, and Aniridia); and glaucoma associated with acquired conditions (e.g., trauma-induced) [2].

All data were fully pseudonymized before they were analyzed. According to the regional laws, the requirement for informed consent was waived by the ethics committee of the medical board of the University Medical Center of Mainz. The study was conducted in accordance with the Declaration of Helsinki.

Patients were identified by systematic examination of the operation protocols and by searching electronic patient history data for matching ICD-10 codes (Q13 and Q15). Identified patients were screened for eligibility, and all records of suitable patients were included.

The following parameters were evaluated and documented: child’s sex; birthdate; age at both glaucoma presentation and intervention; affected eye(s); all ophthalmic diagnoses; and ocular parameters such as IOP, objective refraction, corneal diameter (CD) and corneal status, optic nerve head status, and axial length (AL). The following parameters were recorded at any patient visit: glaucoma medications used (number and type), previous surgical procedures and other ophthalmic surgical procedures performed, surgical complications, IOP, anterior segment ocular features, and refractive status. The axial length, corneal diameter, central corneal thickness, IOP, and optic nerve head status were assessed under general anesthesia. IOP was measured either with Goldmann applanation tonometry (Haag-Streit AG, Koeniz, Switzerland), Perkins applanation tonometry (Kōwa K.K., Nagoya, Japan), or iCare rebound tonometry (Revenio Group Oyj, Vantaa, Finland). When several IOP measurements were obtained per eye, the median value was recorded.

### 2.1. Surgery

Both metal probe trabeculotomy and 360-degree catheter trabeculotomy were performed by three experienced ophthalmic surgeons (F.G., N.P., and E.M.H.). For both procedures, the conjunctiva was opened either at the limbus (limbus-based conjunctival flap in 67 eyes) or 8-mm posterior of the limbus (fornix-based conjunctival flap in 25 eyes), depending on the state of the conjunctiva. A 3.5-mm × 3.5-mm partial thickness scleral flap was dissected. A second, deeper, and smaller flap of 1.5 mm in width and 3 mm in length was formed to unroof the Schlemm’s canal anterior to the scleral spur (as described before by Chin et al. [18]). Before the probe or catheter insertion, acetylcholine (Miochol-E; Bausch & Lomb Inc., Bridgewater, NJ, USA) and a cohesive ophthalmic viscoelastic device (Healon^®^ or Healon^®^GV; Abbott Laboratories Inc., Abbott Park, IL, USA) were injected into the anterior chamber via a paracentesis to achieve miosis and a deep, stable anterior chamber.

For probe trabeculotomy, the probe was then inserted into one ostium of the Schlemm’s canal and gently rotated into the anterior chamber, and this procedure was repeated through the other ostium of Schlemm’s canal. In cases where the opening of Schlemm’s canal was identified with certainty, no gonioscopic control was performed. However, in some patients, especially in secondary glaucoma patients and in anterior chamber and/or angle anomalies, gonioscopic verification was carried out. For 360-degree trabeculotomy, the illuminated iTRACK microcatheter (iTRACK 250A; iScience Interventional, Menlo Park, CA, USA) was inserted and advanced through the entire circumference of the Schlemm’s canal. Once the tip of the catheter appeared in the opposite ostium, both ends were pulled in a purse string manner, thus performing a 360° trabeculotomy. Thereafter, for both procedures, the small, deep, and superficial scleral flaps were closed with single 10-0 nylon lamellar sutures. The conjunctiva was closed either by a 10-0 nylon continuous limbal mattress suture in the cases of fornix-based conjunctival flaps or by a 9-0 vicryl double-layer suture in cases of limbal-based conjunctival flaps.

In cases of combined trabeculectomy with trabeculotomy, the Descemet bridge anterior to the Schlemm´s canal was perforated, and a small trabecular block was removed. Small iridectomy was only performed in the case of iris incarceration.

### 2.2. Outcome Measures

The main outcome measure was long-term IOP development. IOP was recorded by applanation tonometry and by iCare rebound tonometry, if possible, at each visit in both eyes. IOP was analyzed in the following manner: IOP at presentation, at last follow-up or before second operation, and the difference of IOP between presentation and last follow-up or before second operation, as well as the difference in IOP pre- and postoperatively.

Surgical success was defined as IOP < 21 mmHg in eyes without a need for further intervention for pressure reduction. A qualified success was defined as IOP < 21 mmHg requiring pressure-lowering medication. 

The axial length (AL) and corneal diameter (CD) were measured under anesthesia using a-scan ultrasonography. The axial length and corneal diameter were recorded at the first examination and at the last examination. Furthermore, the difference of AL between affected and unaffected eyes was compared over the observation period. 

### 2.3. Statistical Analysis

For categorical data, the absolute and relative frequencies were computed. For continuous data, the median and interquartile range were calculated. The primary (IOP) and secondary outcomes (AL and CD) were compared using the Mann–Whitney *U* test for continuous variables and Wilcoxon signed-rank test for paired analysis. The significance for all analyses was set at *p* < 0.05. The analyses were performed with SPSS version 24 (IBM, Armonk, NY, USA).

## 3. Results

### 3.1. Description of the Population

A total of 61 patients were included: 32 patients had bilateral and 29 unilateral congenital glaucoma, resulting in 93 eyes with childhood glaucoma. Thirty-six children with primary congenital glaucoma (PCG), seven children with Sturge-Weber syndrome, seven children with aphakic glaucoma, six with Axenfeld-Rieger syndrome, three patients with Peter’s anomaly, and two patients with other anomalies were evaluated. The median follow-up time was 78.2 months (interquartile range: 28.8–122.1 months). The mean age at diagnosis was 3.7 ± 5.1 years. Baseline characteristics are described in Table 1. 

Two hundred and seventy-one glaucoma surgeries were performed on all the included eyes (mean 3.3 ± 3.7 interventions per eye), largely due to the high number of cyclodestructive interventions (see Table 2). The approach of surgical management differed for patients with PCG compared to those with other forms of childhood glaucoma. While the proportion of cyclodestructive interventions was 43% in patients with PCG, it was 69% in the others. Accordingly, incisional surgery accounted for 57% for PCG and only 31% for the other forms of childhood glaucoma.

### 3.2. Management and Control of IOP

IOP was obtained at the first and last visits in 84 out of 93 (90%) glaucomatous eyes. Nine eyes with missing data (10%) due to noncompliance of the children at the last visit were excluded from evaluation. Overall, the mean IOP at the first visit was 32.8 ± 10.2 mmHg and decreased to 15.5 ± 7.3 mmHg at the last visit (*p* < 0.001) in all the patients. In the group of PCG patients, IOP from the first visit of 31.2 ± 9.5 decreased to 14.3 ± 5.8 at the last visit (*p* < 0.001).

**Figure 2:**
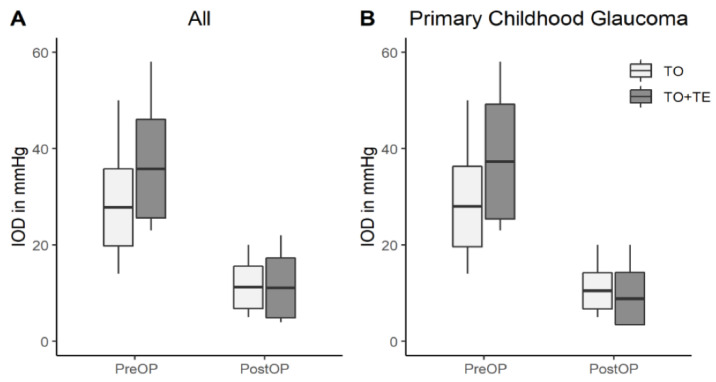
IOP development. IOP values (in mmHg): (**A**) preoperative and postoperative IOP values in all patients (light grey: the metal probe trabeculotomy (TO) group and dark grey: the combined trabeculectomy with trabeculotomy group (TO + TE)) and (**B**) preoperative and postoperative IOP values in the PCG subgroup (light grey: the metal probe trabeculotomy (TO) group and dark grey: the combined trabeculectomy with trabeculotomy group (TO + TE)).

In the metal probe trabeculotomy (TO) group (52 eyes), IOP decreased from 27.8 ± 8 mmHg to 11.2 ± 4.4 after surgery (*p* < 0.001). In the combined trabeculectomy with trabeculotomy group (11 eyes), the preoperative IOP decreased from 35.8 ± 10.2 mmHg to 11.1 ± 6.2 after operation (*p* = 0.003), as shown in Figure 2a. There was no significant difference for IOP after surgery between the two groups (*p* = 0.94). In the subgroup of PCG, 38 eyes were treated with a TO, reducing the IOP from 28.0 ± 8.4 mmHg to 10.5 ± 3.8 mmHg (*p* < 0.001). Furthermore, in the PCG subgroup, seven eyes were treated with combined trabeculectomy with trabeculotomy, reducing the IOP from 37.3 ± 11.9 mmHg to 8.9 ± 5.4 mmHg (*p* < 0.001), as shown in Figure 2b. The PCG subgroup did not show a significant difference in postoperative IOP between the two procedures (*p* = 0.34).

### 3.3. Postoperative Complications

The most common complication after TO was transient hypotony (IOP < 5 mmHg) in six eyes (12%) and IOP spike (2%), suprachoroidal hemorrhage (2%), and pupillary distortion (2%), each occurring in one eye. After combined trabeculectomy with trabeculotomy, transient hypotony was seen in six eyes (55%); four of these (36%) presented choroidal effusion. There was one case of IOP elevation after combined trabeculectomy with trabeculotomy.

### 3.4. Success

At the time of the last follow-up, 62% of all eyes that underwent surgery achieved complete surgical success without IOP-lowering medication. Qualified surgical success (with or without additional medication) was reached by 85% of the eyes. In the subgroup of PCG patients, the rate of qualified surgical success was 89%.

### 3.5. Development of Axial Length (AL) and Corneal Diameter (CD)

The mean AL at the first examination was 22.1 ± 2.2 mm and 23.6 ± 2.9 mm at the last examination. For evaluation of the axial length development, we compared the AL of glaucomatous eyes with the AL of healthy eyes in children with unilateral glaucoma. We did not find a significant difference in axial length growth between glaucoma eyes and normal eyes (*p* = 0.3). When compared, the PCG group showed a significantly higher axial length of 22.6 ± 2.1 mm at the first examination compared to the other forms of childhood glaucoma (21.1 ± 1.8 mm) (*p* = 0.02). At the last examination, this difference was minimized, with an axial length of 23.6 ± 2.5 mm in the PCG group and 23.5 ± 3.6 mm in the other types of childhood glaucoma, respectively (*p* = 0.90). 

The mean horizontal CD at the first examination was 12.7 ± 1.3 mm and 13.2 ± 1.1 mm at the last examination. The CD development was compared between glaucomatous and healthy eyes. Over the observed duration, there was no significant difference in CD growth between glaucomatous eyes and healthy eyes (*p* = 0.80). The mean horizontal CD was significantly larger at the first and last examinations in patients with PCG (13.3 ± 1.0 and 13.6 ± 0.9) compared to patients with other forms of childhood glaucoma (11.6 ± 0.8 and 12.4 ± 1.1) (*p* < 0.001).

### 3.6. Refractive Change

We defined emmetropia as the spherical equivalent within +/−1 dpt, with values below that as myopia and above as hyperopia. Using this definition, 15% of all glaucomatous eyes were emmetropic, 60% myopic, and 25% hyperopic at the baseline. At the end of the study, 16% were emmetropic, 58% myopic, and 26% hyperopic. In healthy contralateral eyes of unilateral glaucoma, 31% showed emmetropia, 8% myopia, and 61% hyperopia at the baseline vs. 54% emmetropia, 23% myopia, and 23% hyperopia at the last examination, respectively. The refractive status of the eyes with PCG and glaucoma of other types is shown separately in Table 3. The rate of myopia was significantly higher in the group of PCG both at the beginning and at the end (*p* < 0.01).

## 4. Discussion

In this study, we evaluated the long-term data of childhood glaucoma in terms of various parameters, such as corneal diameter, refraction, axial length, and intraocular pressure, in 61 children. Furthermore, we analyzed the number and the various types of surgeries performed in children with glaucoma at the University Medical Center, Mainz, Germany. Our study population was heterogeneous with respect to the different forms of childhood glaucoma. The most common entity of childhood glaucoma among our patients was primary congenital glaucoma (PCG), which mirrors its higher prevalence [3,4]; therefore, we paid special attention to the results of this subgroup.

The preoperative IOP in our study cohort was 32.8 ± 10.2 mmHg and was therefore similar to those described by other studies for comparable cohorts of children with glaucoma (29.1–31.5 mmHg) [19,20,21]. We found an IOP reduction of 14.3 ± 5.8 mmHg, which is comparable to the existing literature (values between 11.07 and 17.0 mmHg) [5,21,22]. 

For surgical success, we chose 21 mmHg as our cut-off value, according to the guidelines. However, it should be discussed whether this cut-off-value is sufficient enough for a heterogeneous patient cohort. Most studies have used similar IOP values [5,23], while some authors used higher cut-off values of up to 24 mmHg [24]. The debate about success after glaucoma surgery and its often arbitrarily chosen IOP end points is currently underway, with differing viewpoints concerning the ideal IOP cut-off value [25]. Comparisons of success after glaucoma surgery should therefore take the varying criteria of success into account. We observed a complete surgical success rate, without IOP-lowering medication, of 62% and a rate of 85% for qualified success, where patients with or without IOP-lowering medication were counted as successes. These values are comparable to studies with similar cohorts of patients that also chose a cut-off of 21 mmHg for surgical success, where success rates of 79.5–80.4% were reported [5,26]. However, our somewhat lower success rates can, in part, be explained by the high proportion of secondary childhood glaucoma cases in our population. Patients with secondary childhood glaucoma often have a more advanced disease and do require repeated surgeries, as seen in our cohort. Comparing combined trabeculotomy–trabeculectomy [27,28] vs. trabeculotomy alone, higher preoperative IOP values were present in patients who received the combination operation (35.8 ± 10.2 mmHg) compared to those with standalone trabeculotomy (27.8 ± 8 mmHg). The IOP decreased to almost equal values of 11.2 in the TE + TO group and 11.1 in the TO group, respectively. When only the PCG collective was considered, analogous results were obtained. Khalil et al. compared these two methods in patients with primary congenital glaucoma and reported similar results of 11.1 mmHg for TO and 11.3 mmHg for combined trabeculectomy with trabeculotomy one month after surgery with a baseline IOP of 24 mmHg [29].

The most common surgical side effect was hemorrhage in eyes that underwent trabeculotomy or TO/TE (19.7% and 37.5%, respectively). Since hemorrhage is an expected event in any kind of trabeculotomy, it is not considered a complication. The reported incidences correspond to the values published by other authors (23–34%) [22,29,30,31].

The high count of surgical interventions of 3.3 ± 3.7 per eye is largely due to the high number of cyclodestructive interventions (130 out of 271), which was considerably higher in patients with other forms of childhood glaucoma than PCG (69%) compared to those with PCG (43%). Our cohort received a high number of controlled cyclophotocoagulations. In this modified version of cyclophotocoagulation, laser radiation reflected from the fundus is recorded by a photodetector outside the eye. The time dependence of this detector signal directly monitors the change in transmission of the coagulated tissue; with this information, the surgeon or a computer can interrupt the laser process [32]. The surgical approaches at our clinic have changed over the last ten years. Formerly, cyclodestructive techniques were applied frequently, particularly in secondary glaucoma but, also, in primary congenital glaucoma. This approach has been changed in the last few years, as 360° trabeculotomy and probe trabeculotomy have become the standard of care in childhood glaucoma in our clinic [33,34]. One has to bear in mind that the observation time of this study started in 1995, when other surgical techniques and experience were applied in our clinic. In a similar study, Alsheikheh et al. reported 2.5 interventions per eye during an observation period over 5 years [22]. Zetterberg et al. reported a mean of 2.3 pressure-lowering interventions in an average period of 5.9 years, including a number of cyclophotocoagulations [20].

Various authors consider the axial length an important criterion for the diagnosis and follow-up of childhood glaucoma [35,36,37]. A significant growth of the axial length was found at the first (22.4 ± 2.2 mm) and the last measurements (23.9 + 2.9 mm) of all the eyes, corresponding to physiological elongation. In children with unilateral glaucomatous eyes and healthy eyes, no significant difference in AL growth was seen over the observed period (*p* = 0.3). Kiefer et al. and Mayer et al. reported similar AL values of 22.5 mm preoperatively, 24.2 mm postoperatively and 22.3 mm preoperatively, 23.9 mm postoperatively, respectively [23,26].

Kiskis et al. [38] considered the corneal diameter to be more reliable than the axial length in childhood glaucoma. In our study, however, we could not find a significant difference in corneal diameters between the first and the last visits. Although changes of the corneal diameter may provide some additional information during follow-up of childhood glaucoma, small changes of the corneal diameter are difficult to detect. Thus, axial length changes remain an important morphological parameter in monitoring the progress of glaucoma. Indeed, surgical decisions are not made solely on the basis of IOP but, also, on the basis of AL.

Another important clinical factor is the refractive error [39,40,41]. The baseline prevalence of myopia in our study (60%) corresponds well to other reports [26,42,43,44]. Hyperopia is reported heterogenous in the literature, and the prevalence in our study (25%) corresponds well to the publications by Dannheim [44], Mandel [42], and Meyer [26], who reported prevalences of 22.9, 25.6, and 33.3%, respectively, while Schlieter [43] and Alsheikheh [22] reported lower rates of 11.5% and 0% of hyperopia of more than 1 diopter, respectively. As the refractive error is known to be a valuable indicator of operative success or disease progression [39], the fact that there was no further myopization of the cohort after surgical intervention may be considered positive.

We saw significantly higher myopia rates, corneal diameters, and axial lengths at the first examination in eyes with PCG compared to other forms of childhood glaucoma, which is not surprising considering that buphthalmos is one of the defining diagnostic criteria for PCG. Therefore, myopia, enlarged corneal diameters and axial lengths above the age-appropriate average may be used as indications of PCG in patients with childhood glaucoma.

This study had several limitations. The retrospective study design limited the evaluation of all the clinical parameters; many parameters were only collected when it was therapeutically and diagnostically useful. Moreover, childhood glaucoma, already rare as such, encompasses a wide spectrum of even rarer disorders. Therefore, due to the small number of cases, it was not possible to analyze most subgroups separately, with the exception of PCG. Comparisons between PCG and glaucoma of other types are of limited validity, as the latter group consists of a diverse spectrum of anomalies. However, reasonable data on childhood glaucoma are rare in the literature, and we believe that our data can add some insight into the treatment of glaucoma in newborns and children. 

This study summarizes the treatment of childhood glaucoma over the last two decades until 2015. In conclusion, our results showed the positive results of surgical intervention and underlie the necessity of early and sufficient treatment for childhood glaucoma in specialized centers. However, the treatment has changed since 2015; nowadays, circumferential catheter-assisted trabeculotomy is the main method of choice in our center. 

Since research on childhood glaucoma mainly consists of retrospective studies with smaller cohorts, there is a need for a standardized examination protocol and documentation of pediatric glaucoma, such as a childhood glaucoma database, that could improve the care for children with glaucoma.

## Figures and Tables

**Table 1:** Baseline characteristics.

Characteristic	Total (*n* = 61)
Demographic	
Age, Median (IQR), y	1.0 ± (0.1–7.0)
Female sex % (no.)	42.6 (26)
Bilateral glaucoma % (no.)	52.5 (32)
Preop. IOP Median (IQR), mmHg	31.0 (25.25–38.75)
Medication classes, median (IQR)	3.0 (1.0–4.0)
Classification of childhood glaucoma % (no.)	
Primary congenital glaucoma	59.0 (36)
Glaucoma associated with nonacquired systemic disease or syndrome	
Sturge-Weber syndrome	11.5 (7)
Glaucoma associated with nonacquired ocular anomalies	
Aphakia	11.5 (7)
Axenfeld-Rieger syndrome	9.8 (6)
Peter’s anomaly	4.9 (3)
Glaucoma associated with nonacquired ocular anomalies	3.2 (2)
Haab striae % (no.)	43.0 (40)

IOP = intraocular pressure; IQR = interquartile range; *n* = number.

**Table 2:** Number of operations.

Total Number of Operations	All	PCG
Controlled cyclophotocoagulation	130	61
Cyclocryotherapy	16	5
Trabeculotomy	52	38
Combined trabeculectomy/trabeculotomy	11	7
Revision after trabeculotomy	9	7
Re-trabeculotomy	24	18
Trabeculectomy	2	0
Aqueous Shunt Implantation	19	12
Revision after filtering surgery	8	7

PCG = primary congenital glaucoma.

**Table 3:** Refractive status in eyes with primary childhood glaucoma (PCG) (upper line) and eyes with other forms of childhood glaucoma than PCG (lower line) at the baseline and end of follow-up.

	Baseline			End of Follow-Up		
PCG	Emmetropia	Hyperopia	Myopia	Emmetropia	Hyperopia	Myopia
Yes	8%	18%	74%	18%	13%	68%
No	29%	41%	29%	12%	53%	35%

## Data Availability

The data presented in this study are available on request from the corresponding author. The data are not publicly available due to their containing information that could compromise the privacy of research participants.

## References

[1] Thau A., Lloyd M., Freedman S., Beck A., Grajewski A., Levin A.V. (2018). New classification system for pediatric glaucoma: Implications for clinical care and a research registry. Curr. Opin. Ophthalmol..

[2] Beck A., Chang T., Freedman S., Weinreb R.N., Grajewski A., Papadopoulos M., Grigg J., Freedman S. (2013). Definition, Classification, Differential Diagnosis. Proceedings of the 9th Consensus Meeting: Childhood Glaucoma.

[3] Papadopoulos M., Cable N., Rahi J., Khaw P.T., BIG Eye Study Investigators (2007). The British Infantile and Childhood Glaucoma (BIG) Eye Study. Investig. Ophthalmol. Vis. Sci..

[4] Khan A.O. (2011). Genetics of primary glaucoma. Curr. Opin. Ophthalmol..

[5] Yassin S.A., Al-Tamimi E.R. (2016). Surgical outcomes in children with primary congenital glaucoma: A 20-year experience. Eur. J. Ophthalmol..

[6] Lotufo D., Ritch R., Szmyd L., Burris J.E. (1989). Juvenile Glaucoma, Race, and Refraction. JAMA.

[7] Robin A.L., Quigley H.A., Pollack I.P., Edward Maumenee A., Maumenee I.H. (1979). An Analysis of Visual Acuity, Visual Fields, and Disk Cupping in Childhood Glaucoma. Am. J. Ophthalmol..

[8] Taylor R.H., Ainsworth J.R., Evans A.R., Levin A.V. (1999). The epidemiology of pediatric glaucoma: The Toronto experience. J. AAPOS.

[9] Chen T.C., Chen P.P., Francis B.A., Junk A.K., Smith S.D., Singh K., Lin S.C. (2014). Pediatric glaucoma surgery: A report by the American Academy of Ophthalmology. Ophthalmology.

[10] Morales J., Al Shahwan S., Al Odhayb S., Al Jadaan I., Edward D.P. (2013). Current surgical options for the management of pediatric glaucoma. J. Ophthalmol..

[11] Burian H.M. (1960). A case of Marfan’s syndrome with bilateral glaucoma. With description of a new type of operation for developmental glaucoma (trabeculotomy ab externo). Am. J. Ophthalmol..

[12] Smith R. (1960). A new technique for opening the canal of Schlemm. Preliminary report. Br. J. Ophthalmol..

[13] Chang T.C., Cavuoto K.M. (2013). Surgical management in primary congenital glaucoma: Four debates. J. Ophthalmol..

[14] Dietlein T.S. (2015). Glaucoma surgery in children. Ophthalmologe.

[15] Harms H., Dannheim R. (1969). Erfahrungen mit der trabekulotomia ab externo beim angebore-nen Glaukom. Ber. Dtsch Ophthalmol. Ges..

[16] Papadopoulos M., Edmunds B., Fenerty C., Khaw P.T. (2014). Childhood glaucoma surgery in the 21st century. Eye.

[17] Chang I., Caprioli J., Ou Y. (2017). Surgical Management of Pediatric Glaucoma. Dev. Ophthalmol..

[18] Chin S., Nitta T., Shinmei Y., Aoyagi M., Nitta A., Ohno S., Ishida S., Yoshida K. (2012). Reduction of intraocular pressure using a modified 360-degree suture trabeculotomy technique in primary and secondary open-angle glaucoma: A pilot study. J. Glaucoma.

[19] Bussieres J.F., Therrien R., Hamel P., Barret P., Prot-Labarthe S. (2009). Retrospective cohort study of 163 pediatric glaucoma patients. Can. J. Ophthalmol..

[20] Zetterberg M., Nystrom A., Kalaboukhova L., Magnusson G. (2015). Outcome of surgical treatment of primary and secondary glaucoma in young children. Acta Ophthalmol..

[21] Aponte E.P., Diehl N., Mohney B.G. (2011). Medical and surgical outcomes in childhood glaucoma: A population-based study. J. AAPOS.

[22] Alsheikheh A., Klink J., Klink T., Steffen H., Grehn F. (2007). Long-term results of surgery in childhood glaucoma. Graefes Arch. Clin. Exp. Ophthalmol..

[23] Kiefer G., Schwenn O., Grehn F. (2001). Correlation of postoperative axial length growth and intraocular pressure in congenital glaucoma—A retrospective study in trabeculotomy and goniotomy. Graefes Arch. Clin. Exp. Ophthalmol..

[24] Areaux R.G., Grajewski A.L., Balasubramaniam S., Brandt J.D., Jun A., Edmunds B., Shyne M.T., Bitrian E. (2020). Trabeculotomy Ab Interno with the Trab360 Device for Childhood Glaucomas. Am. J. Ophthalmol..

[25] Rotchford A.P., King A.J. (2010). Moving the goal posts definitions of success after glaucoma surgery and their effect on reported outcome. Ophthalmology.

[26] Meyer G., Schwenn O., Pfeiffer N., Grehn F. (2000). Trabeculotomy in congenital glaucoma. Graefes Arch. Clin. Exp. Ophthalmol..

[27] Mandal A.K., Matalia J.H., Nutheti R., Krishnaiah S. (2006). Combined trabeculotomy and trabeculectomy in advanced primary developmental glaucoma with corneal diameter of 14 mm or more. Eye.

[28] Mandal A.K., Naduvilath T.J., Jayagandan A. (1998). Surgical results of combined trabeculotomy-trabeculectomy for developmental glaucoma. Ophthalmology.

[29] Khalil D.H., Abdelhakim M.A. (2016). Primary trabeculotomy compared to combined trabeculectomy-trabeculotomy in congenital glaucoma: 3-year study. Acta Ophthalmol..

[30] McPherson S.D., Berry D.P. (1983). Goniotomy vs external trabeculotomy for developmental glaucoma. Am. J. Ophthalmol..

[31] McPherson S.D., McFarland D. (1980). External trabeculotomy for developmental glaucoma. Ophthalmology.

[32] Preußner P.-R., Boos N., Faßbender K., Schwenn O., Pfeiffer N. (1997). Real-time control for transscleral cyclophotocoagulation. Graefe’s Arch. Clin. Exp. Ophthalmol..

[33] Hoffmann E.M., Aghayeva F., Schuster A.K., Pfeiffer N., Karsten M., Schweiger S., Pirlich N., Wagner F.M., Chronopoulos P., Grehn F. (2021). Results of childhood glaucoma surgery over a long-term period. Acta Ophthalmol..

[34] Hoffmann E.M. (2020). 360° trabeculotomy for pediatric glaucoma. Ophthalmologe.

[35] Buschmann W., Bluth K. (1974). Regular echographic axial measurements of the eye in controlling intraocular pressure regulation in hydrophthalmos. Klin. Mon. Augenheilkd..

[36] Dietlein T.S., Jacobi P.C., Krieglstein G.K. (1998). Eyeball growth after successful glaucoma surgery in the 1st year of life--follow-up values for primary congenital glaucoma. Klin. Mon. Augenheilkd..

[37] Sampaolesi R., Caruso R. (1982). Ocular echometry in the diagnosis of congenital glaucoma. Arch. Ophthalmol..

[38] Kiskis A.A., Markowitz S.N., Morin J.D. (1985). Corneal diameter and axial length in congenital glaucoma. Can. J. Ophthalmol..

[39] Broughton W.L., Parks M.M. (1981). An Analysis of Treatment of Congenital Glaucoma by Goniotomy. Am. J. Ophthalmol..

[40] Douglas D.H. (1970). Reflections on buphthalmos and goniotomy. Trans. Ophthalmol. Soc..

[41] Haas J. (1968). Principles and Problems of Therapy in Congenital Glaucoma. Investig. Ophthalmol. Vis. Sci..

[42] Mandal A.K., Gothwal V.K., Bagga H., Nutheti R., Mansoori T. (2003). Outcome of surgery on infants younger than 1 month with congenital glaucoma. Ophthalmology.

[43] Schlieter F., Nathrath P., Nicolai R. (1974). Follow-up examination long after surgical treatment of congenital glaucomas (author’s transl). Klin. Mon. Augenheilkd..

[44] Dannheim R., Haas H. (1980). Visual acuity and intraocular pressure after surgery in congenital glaucoma (author’s transl). Klin. Mon. Augenheilkd..

